# Sterile, abscess-like cerebral lesion during trastuzumab therapy after HER2 status switch in a triple negative breast cancer patient: a case report and literature review

**DOI:** 10.1186/s12885-020-07114-7

**Published:** 2020-07-01

**Authors:** Tamás Mezei, Melinda Hajdu, Gábor Czigléczki, Gábor Lotz, Judit Kocsis, Janina Kulka, Anna Horváth

**Affiliations:** 1 Department of Neurosurgery, Semmelweis University, 57 Amerikai street, Budapest, Pest 1145 Hungary; 2 National Institute of Clinical Neurosciences, 57 Amerikai street, Budapest, Pest 1145 Hungary; 3 1st Department of Pathology and Experimental Cancer Research, Semmelweis University, 26 Üllői street, Budapest, Pest 1085 Hungary; 4 2nd Department of Pathology, Semmelweis University, 93 Üllői street, Budapest, Pest 1091 Hungary; 5 3rd Department of Internal Medicine, Semmelweis University, 4 Kútvölgyi street, Budapest, Pest 1125 Hungary

**Keywords:** Triple negative breast cancer, Brain metastasis, HER-2 immunophenotype switch, Trastuzumab, Abscess-like lesion

## Abstract

**Background:**

Breast cancer is a global health problem – it is the most common malignancy among women. Triple negative breast cancers (TNBC) account for 10–20% of female breast cancer. Most TNBC cases confer poor prognosis. Brain metastasis appears in more than 15% in the triple negative breast cancer population, which causes serious decrease in survival. Changes of immunophenotype are not uncommon in breast cancer, offering new therapeutic options in cases where targetable proteins or pathways are being identified.

**Case presentation:**

After five lines of chemotherapy and 82 months following the first diagnosis, our patient with brain metastatic triple negative breast cancer had human epidermal growth factor receptor 2 (HER2) genetic heterogeneity in the metastatic tissue sample interpreted as HER2 status conversion. After the removal of the metastasis, we started first line therapy for metastatic HER2 positive cancer with trastuzumab and paclitaxel. After the first cycle of trastuzumab, on day 8, she had a seizure, and neurosurgical examination showed an abscess-like lesion. The punctate proved to be sterile by microbiological and pathological examination, so we continued cytostatic therapy without the anti-HER2 antibody. 3 months later, we could not identify the previous abscess-like lesion in the control computer tomography (CT) scan, and our patient had no neurological deficits.

**Conclusion:**

We emphasize the importance of regular tissue confirmation of predictive markers in progressive tumorous disease even if our presented case is not unequivocally a “conversion case”. Tumor subtype is determined according to algorithms and definitions published in guidelines, nevertheless, use of different guidelines may lead to controversial interpretation in cases where HER2 genetic heterogeneity is present. Furthermore, we suggest that seronegative, aseptic intracranial fluid effusion after the removal of a brain metastasis may possibly be a side effect of trastuzumab.

## Background

Breast cancer is responsible for the most common female cancer-related morbidity and mortality all over the world [1]. The prevalence of the disease is 23% among women living with cancer. It is the second most common cause of central nervous system (CNS) metastasis. The risk of developing metastasis is 10–16% among those living with breast cancer, and it may be higher in advanced cases with poor prognosis [2, 3]. TNBC is a subgroup of breast cancer, being negative for hormone receptors and HER2. There is a high overlap with the basal-like subgroup of breast cancers characterized by gene expression profiling methods [4–6]. TNBC accounts for roughly one-sixth of all breast carcinomas [7].

A number of studies provided evidence about the instability of hormone receptor and HER2 status during the progression of the disease, with special focus on the relationship of primary tumor and metastases. Alterations occur most frequently in hormone receptor status and Ki67 labeling index, and these changes usually carry a worse prognosis: for example, an estrogen receptor (ER) positive tumor may become negative, and the Ki67 index may increase during the progression of the disease. The triple negative phenotype, and specifically, HER2 negative status is relatively conserved [8].

This report presents the case of a germline breast cancer type 1 (BRCA1) mutation carrier patient with TNBC cancer without androgen receptor (AR) expression, and with a HER2 status switch during the course of the disease. Trastuzumab therapy produced an assumed side effect, which is described here for the first time in the literature.

## Case presentation

Our patient was 32 years old when she first presented at the oncology unit of the 3rd Department of Internal Medicine, Semmelweis University, in July 2010. She had palpated nodules in her right breast. Ultrasonography (USG) and magnetic resonance imaging (MRI) showed bilateral multifocal tumor, with nodules of 8 × 7 mm and 7 × 4 mm located at 6 and 8 o’clock in her right breast, respectively, and an 8 × 5 mm tumor focus located at 5 o’clock in her left breast. Aspiration cytology confirmed the diagnosis of invasive breast carcinoma (no special type). There was no detectable distant metastasis. In her medical history, there was an ovulation induction therapy by clomiphene 3 years before, at age 29, and a childbirth via normal vaginal delivery after that.

**Fig. 1:**
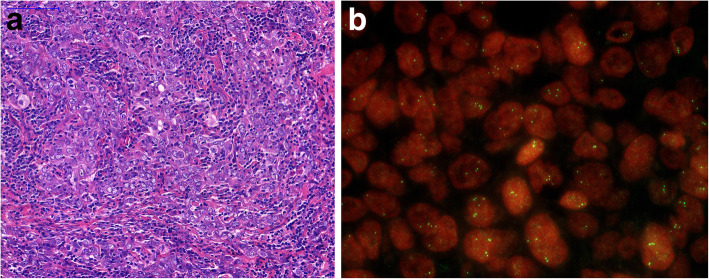
Histopathology from the primary tumor. **a**. Primary ductal invasive breast carcinoma with prominent lymphocytic infiltration (tumor infiltrating lymphocyte (TIL) score was 80–90%), H&E section.**b**. HER2 FISH with single probe: Chr 17 polysomy was diagnosed based on the finding of 3–6 HER2 copies in many tumor cells

The patient underwent bilateral mastectomy with axillary sentinel lymph node biopsy. Pathological examination showed bilateral, grade 3 invasive breast carcinoma (no special type) with ER, progesterone receptor (PR) and HER2 negativity by immunohistochemistry (IHC) and fluorescence in situ hybridization (FISH) (Fig. 1). Disease stage was pmT1b N0(sn) cM0. First line adjuvant chemotherapy was initiated in August 2010, according to the 5-fluorouracil, epirubicin, cyclophosphamide and docetaxel (FEC100-TXT) protocol (3 cycles each), after which she had breast reconstruction surgery twice. Chemotherapy and the other interventions were well tolerated. Chemoterapy doses see schedule (Table 1).

**Table 1:** Summary of our patient therapies and its doses

Surgery	Bilateral mastectomy with axillary sentinel lymph node biopsy	pmT1b N0(sn) cM0
**Adjuvant therapy**	FEC100-TXT	5-Fluorouracyl 500 mg/m2 Cyclophosphamide 500 mg/m2 Epirubicin 70 mg/m2 Docetaxel 100 mg/m2
**1st line**	Bevacizumab+TAX	Bevacizumab 15 mg/kg Paclitaxel 175 mg/m2
**Radiotherapy**	IMRT	54 Gy simultaneous boost was given to the infra-axillary and axillary region and the operated area
**2nd line**	CDDP+TXT	Cisplatin 75 mg/m2 Docetaxel 75 mg/m2
**3rd line**	XEL	Capecitabine 2500 mg/m2
**4th line**	PARP inhibitor	Olaparib 300 mg
**5th line**	VNB	Vinorelbine 30 mg/m2 (only 2 cycle)
**6th line**	Trastuzumab+TAX	Trastuzumab 4 mg/kg Paclitaxel 175 mg/m2
**7th line**	VNB/reinduction	Vinorelbine 30 mg/m2
**8th line**	CMF	5-Fluorouracyl 600 mg/m2 Cyclophosphamide 600 mg/m2 Methotrexate 40 mg/m2

Surveillance MRI in December 2013 detected two lesions indicating disease recurrence: a 10 × 5 mm mass on the outer contour of the implant on the left side, and a paracentral 22 × 28 mm lesion on the right side, the latter involving nearby ribs. USG-guided fine needle aspiration biopsy (FNAB) biopsy demonstrated invasive breast carcinoma (no special type). No repeated immunocytochemical reactions were performed on the sample. Positron emission tomography - computer tomography (PET-CT) showed enhancement in the supraclavicular and parasternal lymph nodes, such that tumor removal was contraindicated. Thus, we started first line bevacizumab-paclitaxel therapy for metastatic TNBC, which was given for 12 cycles. Partial regression was seen in PET-CT after 5 cycles, but surgical intervention was still contraindicated due to the activity seen in the lymph nodes, and chemotherapy was continued. Deoxyribonucleic acid (DNA) sequencing performed during this time period revealed a germline mutation in the BRCA1 gene. Progressive disease was diagnosed by PET-CT at the end of the protocol. Because of the lack of new foci, the patient underwent thoracic surgery in February 2015. Pathological examination of the lymph nodes showed metastases with extracapsular extension and perineural invasion (stage pN3). Predictive markers were not investigated in the surgical specimen. Radiotherapy followed, based on the decision of the multidisciplinary oncoteam: intensity-modulated radiotherapy (IMRT, 54 Gy) and simultaneous boost was given to the infra-axillary and axillary region and the operated area.

In November 2015, multiple lesions (progression in the parasternal lymph node, new pulmonary hilar lymph node involvement and lung metastasis) were visible by PET-CT imaging. We started second line therapy for metastatic TNBC according to the cisplatin, docetaxel (CDDP+TXT) protocol. Control CT imaging performed after 9 cycles showed progression; therefore, the patient was enrolled in a clinical trial, where she received oral capecitabine as third line treatment, and the poly (adenosine biphosphate - ribose) polymerase (PARP) inhibitor olaparib as fourth line therapy.

**Fig. 2:**
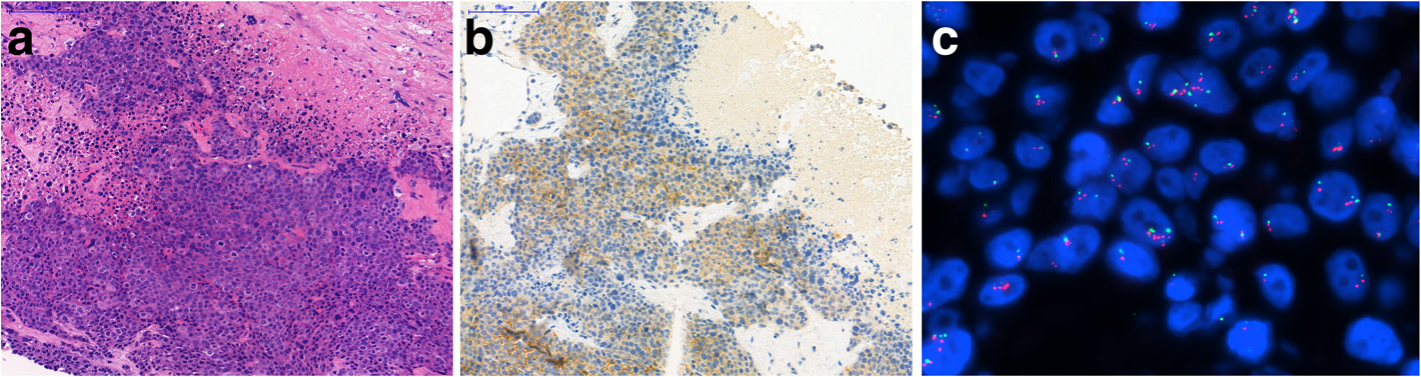
Core biopsy specimen of the tumor mass in the sternum region. **a**. Highly atypical, pleomorphic tumor cells are seen on the H&E section. **b**. HER2 immunohistochemistry **c**. FISH examination confirmed amplification of the HER-2 gene (red) in more than 30% of tumor cells, and chromosome 17 polysomy (green)

Although clinically stable, progression was seen by imaging methods in May 2017 (multiple bone metastases and a new lung metastasis, in addition to parasternal, mediastinal, pulmonary hilar and pelvic lymph node involvement), and the oncological team decided on initiating fifth line therapy (vinorelbine - VNB). Meanwhile, core biopsy was taken from a tumorous mass (34x15mm) above the sternum, and pathological examination confirmed ER- and PR-negative breast cancer involvement, but – surprisingly – HER2 status turned out to be positive by FISH (Fig. 2).

**Fig. 3:**
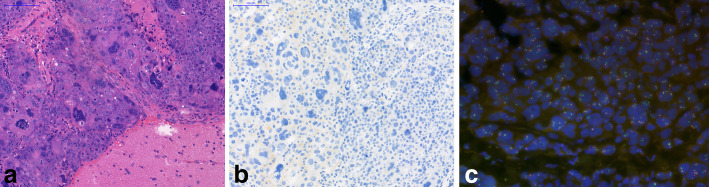
Histopathology of the brain metastasis. **a**. Poorly differentiated tumor cells, some showing very bizarre nuclei, H&E section.**b**. HER2 immunohistochemical reaction heterogeneous positive membrane reaction of tumor cells (20% of tumor cells showed complete, moderately intense membrane reaction).**c**. HER2 FISH: In four different tumor areas 80 tumor cells were counted, the mean *HER2* gene copy number was 4.0/tumor cell, and 1,62/Chr 17. However, 43% of tumor cells showed *HER2* gene amplification with a mean *HER2* gene copy number of 4.6/tumor cell and 2.4/Chr 17. Furthermore, polysomy was identified in 36% of tumor cells with a mean of 3,6 Chr 17/tumor cell. The final conclusion was *HER2* negative status of the metastatic tumor

She developed motor aphasia in June 2017, while at home; she suddenly felt confused, and had a grand mal seizure. She was urgently transferred to the neurosurgery department of the National Institute of Clinical Neurosiences (NICN), and had an MRI scan, which showed a metastasis in the left frontal lobe. Neurosurgical intervention was performed, and the entire tumor was removed, which proved to be the metastasis of TNBC by histopathology (Fig. 3).

Sixth line trastuzumab and paclitaxel treatment was initiated at the end of July – based on the positive HER2 status of the previously sampled sternal mass –, which was given for 2 cycles.

**Fig. 4:**
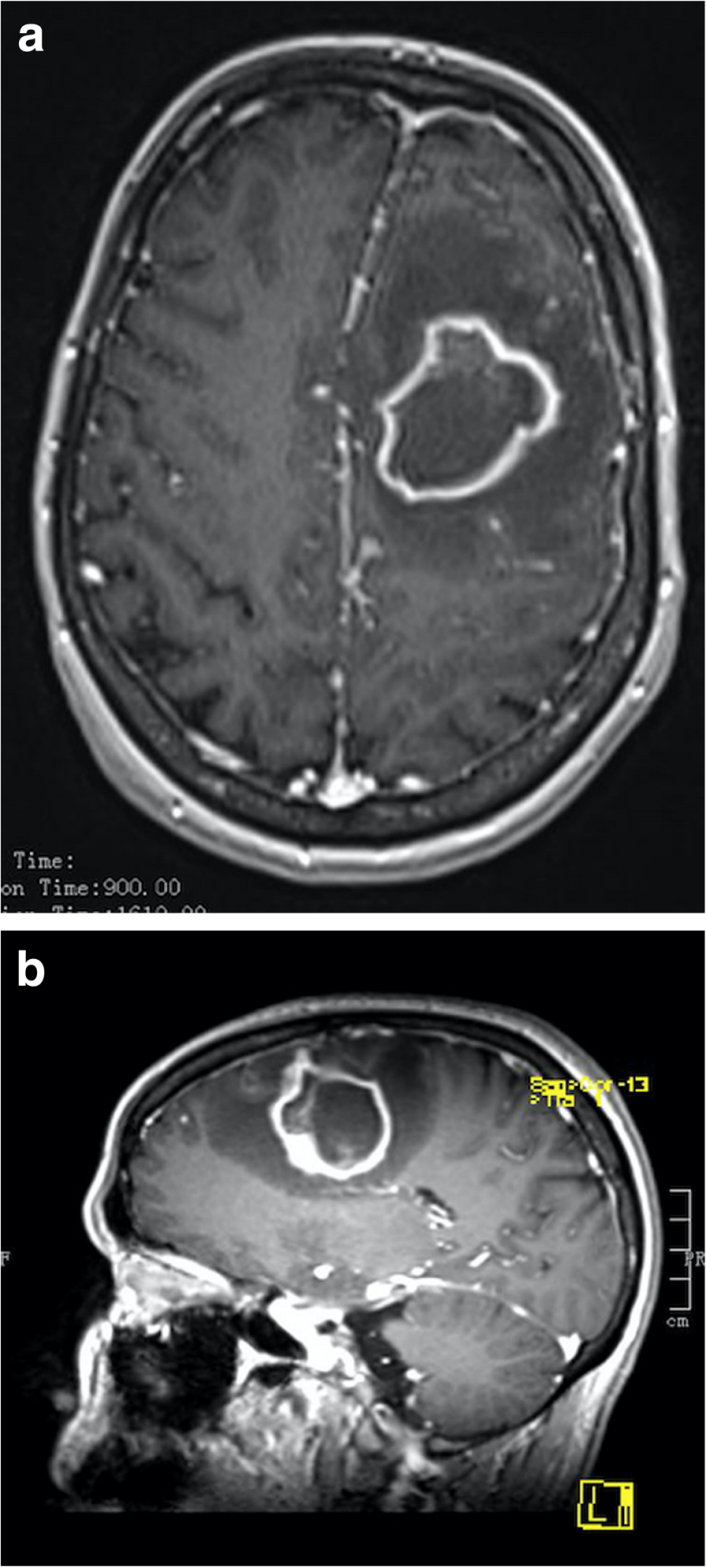
T1-weighted contrast-enhanced horizontal (**a**) and sagittal (**b**) MRI image of the abscess-like cerebral lesion. Ring-enhancing lesion with a central low intensity content and peripheral low intensity, the latter of which is due to the surrounding extensive vasogenic edema

She had a repeated seizure in the middle of August 2017, and she was taken to the NICN. CT and MRI scans showed an abscess-like lesion in the cavity of the previously operated area, surrounded by large perifocal edema (Fig. 4). Mannisole and furosemide was administered for the reduction of intracranial pressure. Stereotactic biopsy was taken on August 09, 2018, and stereotactic drainage was performed on August 29, 2018. During sampling, pus-like content was gained, therefore she received antibiotic therapy (ceftriaxone, vancomycin and metronidazole).

**Fig. 5:**
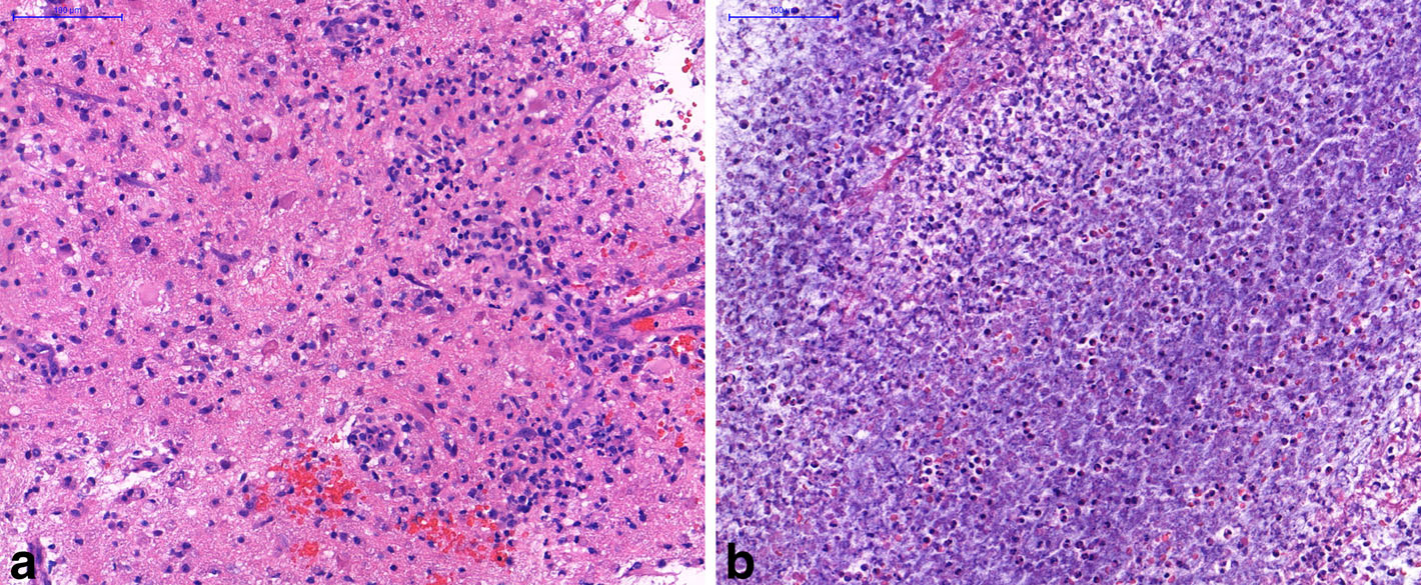
Histopathology from the sampling of the frontal abscess-like lesion. (H&E) Reactive (**a**) and necrotic tissues (**b**) without bacteria or tumor cells, which corresponds to the healing surgical area

Aerobic and anaerobic cultures were negative for bacteria, fungi and parasites as well, and histopathology also excluded the possibility of a true abscess (Fig. 5). After a 30 day pause, she received subcutaneous trastuzumab for the second time, without any side effect.

After seventh line chemotherapy (5 cycles of VNB), control cranial CT showed a new metastasis in the contralateral frontal lobe; the previous abscess-like lesion was not present. The new, right-sided frontal metastasis was treated by stereotactic irradiation.

To be able to decide on further therapy, FISH examination was performed from the intracranial tumor metastasis. It showed HER2 non-amplified status again, and we started eighth line intravenous cytostatic therapy according to the CMF protocol. When she arrived for the 3rd cycle of cytostatic therapy, her performance status dropped (to ECOG 3), and gastric hemorrhage was diagnosed as the cause of weakness. A nasogastric tube was introduced, and the stomach was flushed with acepramine. She received blood transfusion and had a gastroscopy, which identified a gastric ulcer (post-mortem examination later on confirmed the metastatic involvement of the gastric wall). After supportive care was concluded, the patient was placed under hospice care.

She came back to the hospital in very poor general condition (ECOG 4) in December 2017, with symptoms of dehydration, respiratory failure and cardiac decompensation. Intensive therapy was avoided because of her advanced disease. Basic life support and pain relief was provided, and she died without alarming symptoms.

## Discussion and conclusion

The survival curve of TNBC patients shows a decrease between the 3rd and 5th years after first diagnosis; later progression is relatively uncommon [7]. This drop in survival is due to poor prognostic clinicopathological features, such as advanced stage at presentation, unfavorable histopathology, high tumor grade, high Ki67 index, and a higher rate of metastasis [9]. Hazard ratio for relapse in the TNBC group is three times that of the non-TNBC group during this period [7]. Our patient had a similar disease course, with the first relapse occurring 3.5 years after the surgical intervention and adjuvant therapy. Liedtke et al. reported 64% 5 years overall survival in the examined TNBC population [10]. Agarwal et al. reported an 81.8 months median overall survival for TNBC patients [11]. The overall survival of a metastatic TNBC patient is 18 months from the diagnosis of the metastasis [4, 12, 13]. Our patient lived 89 months after her first diagnosis of TNBC (25 months with stage IV disease).

The therapy of TNBC remains a challenge for oncologists treating breast cancer patients [4, 14]. Therapeutic choices include surgical intervention, radiation therapy, systemic chemotherapy and targeted therapy. Our patient underwent bilateral mastectomy as the first step, in accordance with published recommendations for multifocal breast cancer [4]. Adjuvant cytotoxic regimen consisted of anthracyclines and taxanes in her case. The presence of germline BRCA1 mutation allowed for the incorporation of platinum agents into the treatment protocol [4, 15–17], which was utilized as second line therapy. Bevacizumab – an anti-vascular endothelial growth factor (VEGF) antibody inhibiting angiogenesis – was also utilized as part of personalized therapy in the metastatic setting [4]. PARP inhibitors are pharmacological inhibitors of poly-ADP ribose polymerase, an enzyme responsible for repairing single-stranded DNA breaks [18–20]; as they may be given in BRCA mutant cancer, the PARP inhibitor olaparib was included as fourth line therapy. Approximately 25–45% of TNBC express AR, which may be utilized as a therapeutic target, and it is associated with a better prognosis [4, 16, 21]; however, AR status was negative in our case. We used eight lines of systemic therapy and tried every possibility in order to achieve the best quality of life and a longer progression free and overall survival for our patient.

Breast cancer is a heterogeneous disease, and immunophenotypic changes may occur during progression. As the disease progresses, hormone receptor (especially PR) positivity is usually lost and mitotic activity increases [22, 23]. HER2 conversion is also known in the literature, but it is less frequent than the other phenotypic changes [24]. If phenotypic changes in metastases are not searched for, the patient may not receive adequate treatment in the metastatic setting; therefore, the biopsy of the metastatic lesions is recommended [24–27], such as in our case.

Changes in tumor subtype during progression of breast cancer has gained major attention in the recent decade. The largest meta-analysis to date found an overall change in ER receptor status in 19.3%, in PR receptor status in 30.9% and changes in HER2 status in 10.3% [28]. In our case, however, the HER2 status change might not have occurred purely as a biological phenomenon (e.g. clonal selection, or accumulating further mutations, etc.) during disease progression. As Table 2. shows, interpretation guidelines for HER2 IHC and FISH changed considerably during the course of the patient’s disease [29, 30]. Furthermore, in our case, both the primary tumor and its two investigated metastases showed HER2 heterogeneity, both at protein expression level and at the genetic level. Interpretation guidelines for HER2 genetic heterogeneity are not fully unequivocal. The last guideline dealing especially with this problematic field dates from 2009 [31], and defines HER2 (scattered) genetic heterogeneity as 5–50% of tumor cells showing HER2 amplification. In such cases the mean HER2 copy numbers and the mean HER2/chromosome enumeration probe 17 (CEP17) ratio (counted in at least 60 tumor cells) would define the final HER2 status. The tumor can be interpreted as HER2 positive – and thus eligible for targeted therapy – only if the ratio of amplified tumor cells exceeds 50%. The situation is different when there is a well identifiable separate clone present as a cluster of HER2 amplified tumor cells. The rule for interpretation in such cases is that if the cohesive cluster of HER2 amplified cells exceeds 10% of the tumor area investigated, HER2 positive status is to be reported. However, different ideas were published by opinion leaders in the following years: Hanna and co-workers [32] suggested an algorithm for the interpretation of scattered HER2 genetic heterogeneity different from that of Vance and members [31]: according to their suggestion, even in cases of scattered amplified tumor cells, HER2 amplified status can be assigned in cases where the ratio of amplified cells exceeds 10%. In our case, HER2 status of the primary tumor was interpreted as negative according to the 2007 American Society of Clinical Oncology/College of Amercian Pathologists (ASCO/CAP) guideline. The first investigator of the metastasis that occurred in 2017 in the sternum region, interpreted HER2 status as positive using Hanna’s criteria, while the HER2 status of the brain metastasis occurring in the same year - and analyzed in another pathology department - was interpreted as negative using Vance’s and CAP’s criteria.

**Table 2:** Interpretation guidelines for HER2 IHC and FISH changes in our case

YEAR	LESION/MATERIAL EXAMINED	HER2 IHC	HER2 FISH	Respective ASCO/CAP GUIDELINE definition for HER2 positive IHC result	Respective ASCO/CAP GUIDELINE definition for HER2 positive ISH result
**2010**	Primary tumor surgical resection specimen	20% of tumor cells showed complete, intense circumferential membrane reaction: 2+ (SP3 antibody)	Polysomy of Chr17 suggested: many tumor cells showed 3–6 HER2 copies/cell **Interpreted as HER2 negative** (single probe ISH assay)	***2007 ASCO/CAP guideline*** > 30% of tumor cells show complete, intense circumferential reaction	***2007 ASCO/CAP guideline*** > 6 *HER2*copy/cell (single probe ISH) > 2.2 *HER2*/*CEN17* ratio/cell (dual probe ISH)
**2017**	Metastasis (sternum region)	20% of tumor cells showed complete, weak or moderate, circumferential membrane reaction: 2+ (4B5 antibody)	30% of tumor cells showed polysomy-co-amplification (3–6 CEP17 signals and 6–10 HER2 signals/cells; dual probe ISH assay) **Interpreted as HER2 positive**	***2013 ASCO/CAP guideline*** > 10% of tumor cells show complete, in tense circumferential reaction	***2013 ASCO/CAP guideline*** See: Wolff AC et al. Arch Pathol Lab Med. 2014 Feb; 138 (2): 241–256.
**2017**	Metastasis (brain – analysis following second opinion request)	15–20% of the tumor cells showed complete, weak or moderate, circumferential membrane reaction: 2+ (4B5 antibody)	80 tumor cells were counted: mean HER2 copy number/cell was 4.0, mean HER2/CEP17 ratio was 1.62. However, scattered, heterogeneous amplification was present: In 43% of the tumor cells 4.6 HER2/cell was found and the HER2/CEP17 ratio was 2.4. (dual probe ISH assay) **Interpreted as heterogeneous amplification, HER2 negative**	***2013 ASCO/CAP guideline*** > 10% of tumor cells show complete, intense circumferential reaction	***2013 ASCO/CAP guideline*** and ***2009 CAP guidelines for genetic heterogeneity in HER2 testing:*** „HER2 genetic heterogeneity (GH) exists if there are more than 5% but less than 50% of infiltrating tumor cells with a ratio higher than 2.2 …. If more than 50% of the infiltrating tumor cells have a ratio higher than 2.2, then the tumor is considered HER2 amplified.”

Brain metastases occur more frequently in younger women, in the case of poor prognostic markers, such as TNBC and high-grade tumors. The most common symptoms are headache, nausea, vomiting, hemiparesis and visual disturbances; less frequently, seizures. More than half of the metastases are supratentorial, and approximately 25% are localized in the frontal lobe [3]. The appropriate treatment option for a patient with a solitary metastasis and good performance status is the surgical approach [33]. The rate of postoperative complications does not increase with en bloc resection compared to piecemeal resection; on the contrary, Patel et al. published their overall complication rates: 13% with en bloc resection and 19% with piecemeal resection (the probability of infections is under 1%) [34]. The overall survival after brain metastasis develops in a TNBC patient is really poor, but it can be prolonged with personalized systemic therapy. Our patient survived for 7 months after the detection of the first cerebral lesion, and seizures were the initial symptoms of the metastasis.

Remarkably, 82 months after the first diagnosis of TNBC, the biopsy taken from a metastatic sternal mass was interpreted as HER2 positive, which provided the opportunity for starting biological therapy for HER2 positive breast cancer following surgical removal of the brain metastasis (our choice of immunotherapy was trastuzumab, because at that time, pertuzumab treatment was only defrayed by the Hungarian National Health Insurance Fund in the first-line therapy of the breast cancer patients). Trastuzumab treatment was well tolerated, but the patient presented with repeated seizures after 6 weeks, and MRI scan showed an abscess-like cerebral lesion. However, no new metastasis was detected, and we assumed that the abscess-like sterile effusion in the operated area could be a side effect of trastuzumab. After 30 days she got subcutaneous trastuzumab for the second time, without any side effect. Although we suggest that seronegative, aseptic intracranial fluid effusion after the removal of a brain metastasis may possibly be a hitherto undescribed side effect of iv. trastuzumab, we also assume that subcutaneous administration may be safe, as was in our case.

General complications of trastuzumab therapy are well known. It may cause a flu-like syndrome (similarly to other immunological therapies), which is relatively rare because of the humanized nature of the monoclonal antibody. The major problem with trastuzumab therapy is cardiotoxicity. Cardiac dysfunction is primarily characterized by cardiomyopathy – most often as an asymptomatic decrease in left ventricular ejection fraction, and less frequently as congestive heart failure [35]. Trastuzumab combined with taxanes or vinorelbine has also been reported to cause general fluid retention and pleural effusion, which may also represent symptoms of heart failure [36].

Interestingly, despite the HER2 positive, rapidly proliferating metastatic sternal mass, the brain metastasis appearing later had an ER, PR and HER2 negative status; therefore, the possibility of subsequent lapatinib therapy was ruled out.

Taken together, our case teaches us humility: even in the era of advanced molecular genetic diagnostic methods and major breakthroughs in targeted therapy, imperfections in HER2 diagnostic/interpretation methods may be painfully tangible in certain cases. Unequivocal guidelines (and their unanimous use in practice) for the interpretation of HER2 genetic heterogeneity are mandatory.

## Data Availability

The datasets used and/or analysed during the current study are available from the corresponding author on reasonable request.

## Abbreviations

AR: Androgen receptor
ASCO: American Society of Clinical Oncology
BRCA1: Breast cancer type 1
CAP: College of Amercian Pathologists
CDDP: Cisplatin
CEP17: Chromosome enumeration probe 17
CMF: Cyclophosphamide, methotrexate and 5-fluorouracil
CNS: central nervous system
CT: Computer tomography
DNA: Deoxyribonucleic acid
ER: Estrogen receptor
FEC100: 5-fluorouracil, epirubicin and cyclophosphamide
FISH: Fluorescent in situ hybridization
FNAB: Fine needle aspiration biopsy
HE: Hematoxylin-eosin
HER-2: Human epidermal growth factor receptor 2
IHC: Immunohistochemistry
IMRT: Intensity-modulated radiotherapy
MRI: Magnetic resonance imaging
NICN: National Institute of Clinical Neurosciences
PARP: Poly (adenosine biphosphate - ribose) polymerase
PET-CT: Positron emission tomography - computer tomography
PR: Progesterone receptor
TAX: Paclitaxel
TIL: tumor infiltrating lymphocyte
TNBC: Triple negative breast cancer
TXT: Docetaxel
USG: Ultrasonography
VEGF: Vascular endothelial growth factor
VNB: Vinorelbine
XEL: Capecitabine
